# Durability analysis of rotary direct drive electro-hydraulic pressure servo valve based on failure physics principle

**DOI:** 10.1371/journal.pone.0316711

**Published:** 2025-02-21

**Authors:** Jianrui Zhang, Xing Wei, Ni Li, Xiaonan Pan, Yitong Sun

**Affiliations:** 1 College of Intelligent Manufacturing, Longdong University, Qing yang, China; 2 Operation Office of Production Capacity Construction Project Team of No. 10 Oil Production Plant, Changqing Oilfield Company, Qing yang, China; University of Sharjah, UNITED ARAB EMIRATES

## Abstract

The rotary direct drive electro-hydraulic servo valve (RDDPV) is extensively employed in hydraulic systems across aerospace, automotive, and various industrial sectors owing to its remarkable precision and rapid response characteristics. The investigation of the durability life of such devices, constituting intricate amalgamations of mechanical, electrical, and hydraulic components, has perennially posed a formidable challenge. To address this challenge, our study proposes a methodology grounded in failure mechanisms to systematically quantify the durability life of RDDPV. In conjunction with finite element analysis, this study delves into the fatigue durability of the transmission mechanism and the wear durability of the slide valve—two components recognized as vulnerabilities within the RDDPV. Initially, a novel approach is proposed that integrates probability theory and fuzzy theory with the traditional Miner theory, enhancing the accuracy of fatigue life predictions for transmission mechanisms. Subsequently, a meticulous examination of the wear mechanism of the slide valve ensued, wherein we quantitatively characterized the radial wear between the valve core and sleeve using the degree of line wear. Ultimately, employing durability index calculations, the total operational life of the valve is ascertained at about 435,000 hours, thereby aligning with national standards. This research methodology not only contributes significantly to the field but also holds substantial reference value for the precise quantification of the durability life of analogous electro-hydraulic pressure servo valves.

## 1. Introduction

In the 1990s, a novel rotary direct-drive electro-hydraulic servo valve structure emerged, PDDPV [1–3]. This servo valve translates torque motor rotary motion into linear motion through a rotary drive mechanism, eliminating the traditional hydraulic amplifier stage found in conventional servo valves. Instead, it incorporates electric feedback servo control, resulting in a straightforward structure, high reliability, resistance to pollution, and a flexible control mode. Moreover, the arrangement of rotary direct-drive servo valves is more compact compared to linear direct-drive valves, as the motor rotation and the slide valve’s translation are perpendicular [4]. This design has gradually found applications in foreign countries, particularly in aircraft electronic anti-skid brakes, aircraft rudder control, engine control, and related fields [5].

As a typical electro-hydraulic servo valve, RDDPV is a key component in the servo control system and has a decisive impact on the operating characteristics of the system and even the entire machine [6]. Combining electromechanical and hydraulic precision control elements, the electro-hydraulic servo valve involves a complex energy conversion process and intricate parameter-matching relationships [7]. Consequently, the analysis and enhancement of stability and rapid response performance of electro-hydraulic servo valves have become prominent areas of research interest both domestically and internationally. Wang et al. [8] investigated the causes of whistling in nozzle baffle amplifiers and proposed a method to mitigate vibration-induced whistling by adjusting parameters related to the return damping element. Additionally, previous studies [9, 10] delved into the impact of air pockets and return pressure fluctuations on servo valve stability, while Ni et al. [11] analyzed the influence of electronic controller parameters on the dynamic characteristics of electro-hydraulic servo systems. Kang et al. [12] derived a mathematical model of the nozzle baffle pressure servo valve and performed three-dimensional modeling and simulation analysis of the transient flow dynamics on the baffle. Zhang et al. [13] utilized a combination of theoretical derivation and numerical simulation technology to comprehensively analyze the jet tube servo valve, scrutinizing both individual components and the system as a whole. Li et al. [14] proposed an RDDPV valve driven by a stepper motor. By analyzing its working principle, a steady-state mathematical model reflecting the relationship between pressure, flow rate, and angular displacement is derived. Notably, pressure servo valves, due to their significant output gain, present pronounced issues with vibration and instability [15].

Liang et al. [16] presented an approach for evaluating degradation in performance and predicting the erosion lifespan of the DJSV at different levels of oil pollution. the performance degradation of the whole valve was examined, thereby predicting the erosion life of the valve. In a related study, Zhang and Yao et al. [17] focused on assessing performance degradation and predicting the life expectancy of electro-hydraulic servo valves in the presence of wear. Their analysis delved into wear mechanisms utilizing AMESim and Computational Fluid Dynamics (CFD) software. Additionally, Huang et al. [18] undertook an evaluation of performance degradation and life prediction concerning dual-hydrogen servo valves, emphasizing physical failure modes amidst wear conditions. They, too, utilized Computational Fluid Dynamics (CFD) software to assess performance degradation and predict the life span of electro-hydraulic servo valves in such conditions. Moreover, Liu et al. [19] performed simulation analyses based on the physical failure mode of the dual-nozzle electro-hydraulic servo valve. Concurrently, they designed a wear test system for predicting servo valve wear life, yielding promising outcomes. Furthermore, Zhang et al. [20] employed a combined approach integrating CFD and erosion theory to simulate and analyze wear durability in electro-hydraulic servo valves exposed to contamination. Their findings revealed erosion wear near the sharp edges of the valve sleeve and spool, establishing a nonlinear relationship between wear rate and slide valve opening.

As a multifaceted component amalgamating mechanics, electronics, and hydraulics, contemporary investigations into the dynamic and static performance of electro-hydraulic servo valves have achieved a commendable level of maturity. Conversely, scrutiny into aspects of reliability and enduring functionality, particularly extended operational lifespans encompassing fatigue and wear, is comparatively underdeveloped. Limited attention has been devoted to comprehensively studying the durability life of cycles, with existing research predominantly fixated on localized or singular failure modes, such as wear failure attributable to contaminant particles. Moreover, scant efforts have been made towards predicting the entirety of valve life based on diverse failure mechanisms. Addressing this research gap, our study advocates a durability analysis methodology rooted in failure mechanisms. This approach systematically considers the fatigue failure of two vulnerable components within the RDDPV, namely the transmission mechanism, and the wear failure of the slide valve, thereby facilitating a quantitative exploration of the RDDPV’s durability life. The primary contributions of this research encompass:

1. Proposing a novel method that integrates probability theory, fuzzy theory, and traditional Miner theory to predict life loss under the fatigue limit;
2. Employing the linear wear degree to quantitatively analyze radial wear between the slide valve sleeves concerning wear;
3. Conducting a quantitative study on the durability life of RDDPV, accounting for both fatigue and wear failures. These research methodologies bear significant reference value for qualitative analysis, durability prediction modeling, and the quantitative assessment of service life in analogous electro-hydraulic servo valves.

The subsequent chapters unfold as follows: Chapter 2 elucidates the working principle of RDDPV and establishes the durability failure threshold. Chapter 3 models and analyzes the fatigue failure of the transmission mechanism, while Chapter 4 addresses the slide valve wear model and analysis. Chapter 5 presents the analysis results and ensuing discussions, culminating in Chapter 6, which offers a comprehensive summation of this research.

## 2. Working principle of RDDPV valve

**Fig 1:**
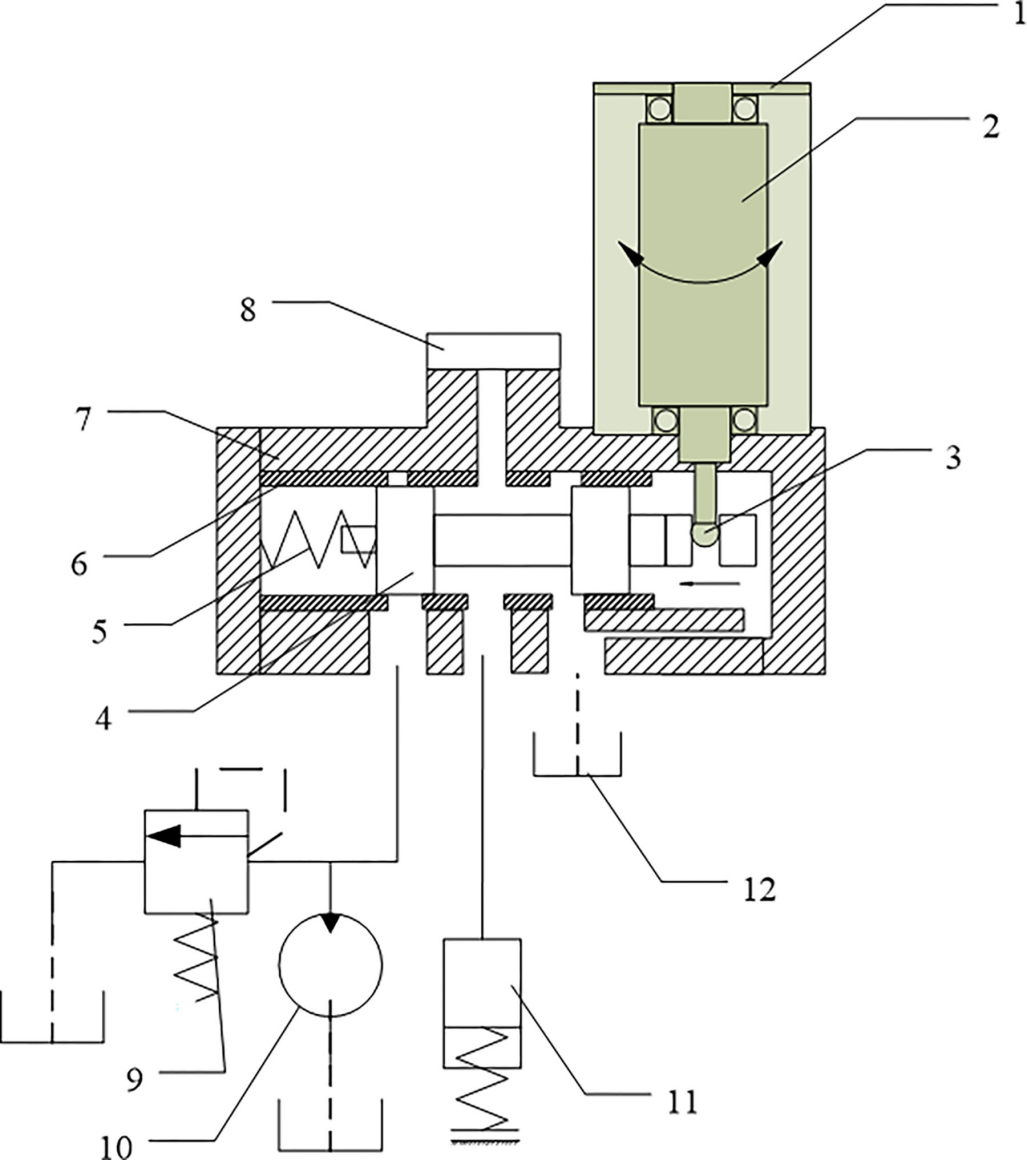
Schematic diagram of the working principles of RRDDPV. 1. Angular displacement sensor;2. Finite torque motor;3. Eccentric drive mechanism; 4. Power slide valve;5. bias spring;6. valve sleeve;7. valve body;8. pressure sensor; 9. overflow valve;10. hydraulic pump;11. Load cavity; 12. oil box.

The working principle of rotary direct drive electro-hydraulic pressure servo valve (RDDPV) is illustrated in Fig 1, and it mainly consists of an electronic controller, limited angle torque motor, eccentric drive mechanism, slide valve pair, and related sensors. When the input command of the electronic controller is 0, the limited angle torque motor has no torque output, at this time, the slide valve is pushed to the far right end by the reset spring, the oil inlet is closed, the working chamber and the oil return port are connected, and the output pressure of servo valve is zero; when a non-zero positive command current signals is input, the electronic controller performs calculation and outputs a PWM signal to drive the limited angle torque motor to rotate, and the eccentric drive mechanism converts the rotational motion of motor into the linear motion of power spool; the power slide valve is in the form of under lap, and the linear movement of spool changes the throttling area ratios of the oil inlet and return ports; the load cavity connected to the oil outlet is a closed sealed cavity during braking, so the steady-state pressure of load cavity only changes with the throttling area ratios of the oil inlet and return ports; the effective stroke of spool is the amount of under lap [21]. The valve adopts the form of electric feedback for servo control; the angle displacement sensor feeds back the rotation angle of torque motor to the sensor to form a closed-loop of motor position; the pressure sensor feeds back the pressure of the working chamber to provide external closed-loop control of pressure [22].

The rotary direct drive electro-hydraulic pressure control servo valve plays a crucial role in modern hydraulic systems by precisely regulating the flow and pressure of hydraulic fluids. These valves are widely used in various applications, including aerospace, automotive, and industrial sectors, where accurate and reliable control of hydraulic systems is essential. The purpose of this research is to investigate and analyze the durability aspects of the rotary direct drive electro-hydraulic pressure control servo valve. Specifically, we aim to identify potential weak points within the valve assembly that may affect its durability and performance. By conducting a fatigue durability analysis of the transmission mechanism and wear durability analysis of the slide valve, we aim to assess the service life and reliability of the valve under different operating conditions. Understanding the factors influencing the durability of the rotary direct drive electro-hydraulic pressure control servo valve is of paramount importance for ensuring its long-term functionality and preventing unexpected failures. The insights gained from this research will contribute to the development of improved design guidelines, maintenance strategies, and performance optimization techniques for these critical components in hydraulic systems. By addressing the research objectives outlined above, we aim to enhance the overall understanding of the rotary direct drive electro-hydraulic pressure control servo valve and contribute to advancements in hydraulic system design, efficiency, and reliability.

**Fig 2:**
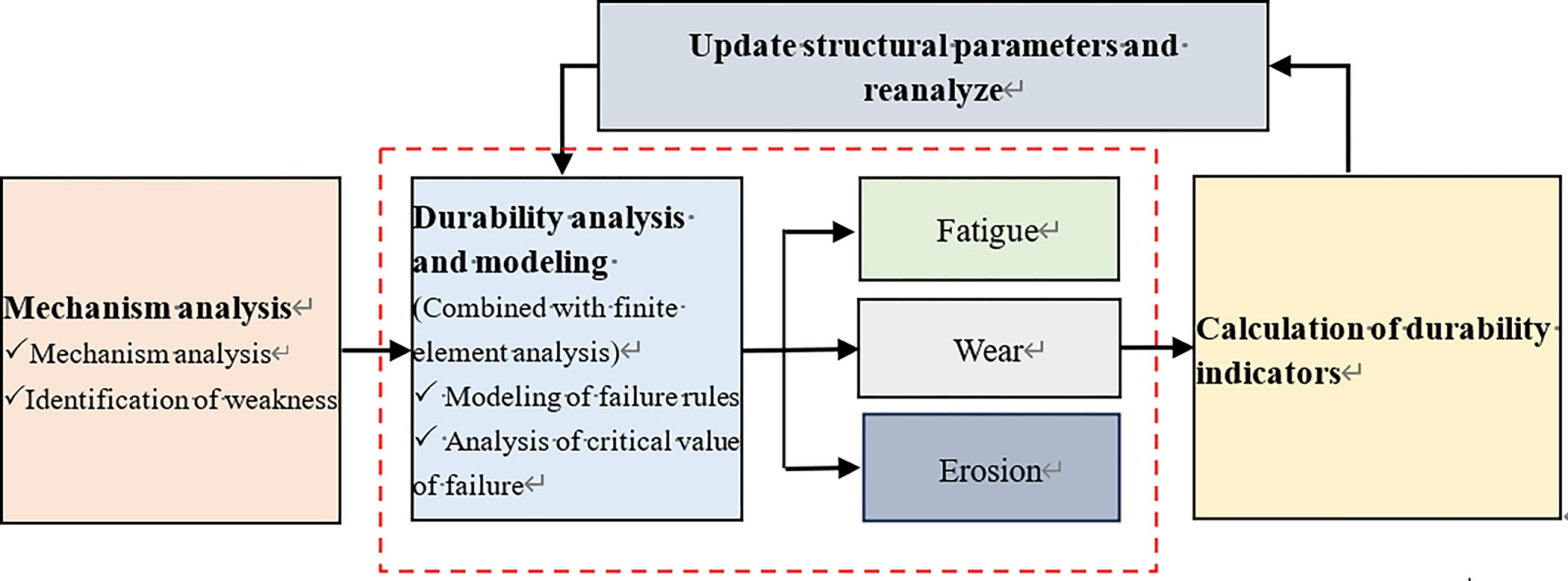
Composition of durability analysis method based on the physics principles of failure.

Product durability refers to its ability to fulfill specified functions until it reaches the failure state under predetermined conditions of use, storage, and maintenance. Traditional test methods for product durability are unsuitable for complex electromechanical systems due to their high cost and low efficiency [23]. To address this issue, we utilized advanced computer tools to conduct tests and analyses based on the principles of failure physics [24]. In this study, we present a system durability analysis method based on the principles of failure physics for analyzing the durability and predicting the service life of complex components such as RDDPV (Fig 2). Firstly, an analysis of the working principles of intricate components is conducted, identifying inherent weak links. Secondly, the endurance life of the weak link is analyzed based on the failure physical model of fatigue, wear, and erosion, etc., which can be combined with the finite element analysis software. Finally, a holistic calculation of the durability index for the entire component is executed, enabling an assessment of its compliance with design requirements. In cases where the durability index falls short, structural parameters are optimized, prompting a re-analysis of the weak links’ durability until the prescribed product requirements are met. Additionally, we employ targeted models for simulation and calculation to carry out the durability and service life prediction. The durability analysis of RDDPV includes (1) Fatigue durability analysis of the transmission mechanism; and (2) Wear durability analysis of the slide valve.

Initially, Subsequently, the endurance life of these weak links is examined using a comprehensive failure physical model encompassing factors such as fatigue, wear, and erosion. This analysis is augmented by employing finite element analysis software such as Fluent.

Currently, there is no standardized criterion to determine the failure of the RDDPV [25]. In practical applications, the regulation time *t*_*s*_ and the overshoot *σ*% are often used to reflect the dynamic performance of the system, and the steady-state error *E*_Δ_ is used to represent the steady-state performance of the system. In reality, there are various types of systems, and there are no uniform requirements for system indicators. In this section, *α*_*s*_ is set as the multiple of the required initial adjustment time *t*_*s*_, that is, with the degradation of system performance, if the adjustment time is longer than *α*_*s*_*t*_*s*_, it is considered a system failure; *β*_*σ*_ is the required overshoot indicator value; *γ*_*E*_ represents the ratio of the required steady-state error *E*_Δ_ to the expected output of the system. Then, the critical values of the parameters that could cause failure of various subsystems can be determined according to Eq (1). *α*_*s*_ = 2, *β*_*σ*_ = 25% and *γ*_*E*_ = 20% are selected to determine the critical values of the parameters when the valve system fails.

$$P_{ara}=\{\begin{array}{l} max(P_{\alpha_{s}},P_{\beta_{\sigma }},P_{\gamma_{E}}),P\;gradually\;decreases\\ min(P_{\alpha_{s}},P_{\beta_{\sigma }},P_{\gamma_{E}}),P\;gradually\;increases \end{array}$$ (1)

where, *P*_*ara*_ represents the critical value of parameter for valve failure. $P_{\alpha_{s}}$ represents critical value of the parameter for valve failure determined according to the adjustment time. $P_{\beta_{\sigma }}$ represents the critical value of parameter for valve failure determined according to the overshoot. $P_{\gamma_{E}}$ represents the critical value of parameter for valve failure determined according to the steady-state error.

The main structural parameters of RDDPV are listed in Table 1.

**Table 1:** Main structural parameters of RDDPV.

Parameter	Value	Parameter	Value
*t*_*e*_ /ms	1.6	*k*_*t*_/N•m•A^-1^	0.05
*D*_*v*_/mm	6	*U*/m	0.1
*m*_*v*_/g	5	*J*_*βv*_/g•cm^2^	16
*x*_*v0*_/mm	1	*k*_*v*_/kN•m^-1^	6
*h*/mm	1.6	*e*/mm	1.2
*R*_*c*_/Ω	23.3	*V*/ml	10

*t*_*e*_ is the motor mechanical constant, *D*_*v*_ is the diameter of the spool end face, *m*_*v*_ is the spool mass, *x*_*v0*_ is the pre-compression of the reset spring, *h* is the distance from the center of the ball to the plane, *R*_*c*_ is the motor internal resistance, *k*_*t*_ is the current moment coefficient, *U* is the slide valve pre-opening, *J*_*β*v_ is the spool rotational inertia, *k*_v_ is the reset spring stiffness, *e* is the eccentricity distance between the eccentric shaft and the motor rotation center, *V* is the load chamber volume.

This paper analyzes the fatigue durability of the RDDPV transmission mechanism and the wear durability of the spool valve. It investigates the failure mechanisms that impact the potentially weak components of the jet tube servo valve’s durability. Software simulations and durability life calculations are conducted to analyze these aspects. By utilizing software simulations and theoretical calculations, the durability life of the potential weak components of the electro-hydraulic pressure servo valve is determined.

## 3. Fatigue durability analysis of transmission mechanism

The torque motor assembly of the RDDPV comprises a limited angle motor and an eccentric drive mechanism. Upon receiving the control signal, the drive motor undergoes rotary motion, propelling the rotation of the eccentric drive mechanism [26]. The ball located at the drive mechanism’s end experiences the reaction torque exerted by the spool. Throughout the movement of the drive mechanism, a cyclic alternating stress with a stress ratio of R = -1 occurs at the transition joint between the ball and the motor. After a certain number of cycles, the drive mechanism is susceptible to cracking or fatigue fracture. The quantity of alternating stress cycles corresponds to the durability and fatigue life of the valve.

### 3.1 Durability analysis and modeling

#### 3.1.1. Analysis of failure critical value

The stiffness value of the eccentric actuator rod has a greater impact on the performance of the front stage, which in turn affects the performance of the entire pressure servo valve. Based on the static-dynamic part of the simulation results, it is known that the spring tube stiffness decreases by 15% as the critical value for the fatigue life calculation [27].

#### 3.1.2. Modeling of failure rules

Fatigue failure is commonly regarded as a process of gradual accumulation, and consideration of cumulative damage has always been pivotal in finite life design [28]. As per the relevant guidelines, the widely employed theories for cumulative fatigue damage encompass linear fatigue, bilinear fatigue, and nonlinear fatigue cumulative damage. However, the latter two theories are typically avoided in practical projects due to their intricate theoretical formulas and numerous parameters. Conversely, the linear cumulative damage theory, known as the Miner theory, is extensively used in fatigue life prediction owing to its simplicity.

Assume that the loading process of a test piece includes *r* different stress levels *σ*_1_, *σ*_2_,⋯,*σ*_*i*_,⋯,*σ*_*r*_. If *n*_1_, *n*_2_,⋯,*n*_*i*_,⋯,*n*_*r*_ represent the number of cycles at each stress level during fatigue failure, while *N*_1_, *N*_2_,⋯,*N*_*i*_,⋯,*N*_*r*_ represent the life of the test piece when failure occurs only under the effect of the stress level, the basic expression of Miner theory can be obtained as in Eq (2).

$$D=\sum_{i=1}^{r}\frac{n_{i}}{N_{i}}$$ (2)

where, *D* represents damage of test piece, when *D* = 1, the test piece is considered as damaged.

The Miner theory include the following basic assumptions:

a. When the stress level is greater than the material fatigue limit, it will cause damage to the test piece. The damage caused by the stress smaller than the fatigue limit can be ignored, and the service life of the test piece under such stress can be considered as infinite.

b. When the total damage caused by various levels of stress is 1, the sample is damaged.

c. Under a given level of stress, the fatigue life of the test piece is independent of the loading sequence.

The traditional Miner theory fails to consider fatigue dispersion and the damage caused by stress below the fatigue limit, leading to significant errors in predicting the fatigue life of the test piece. To overcome this limitation, this section combines probability theory and fuzzy theory with the traditional Miner theory to predict the test piece’s fatigue life. The prediction model consists of two parts: fatigue dispersion modeling and damage modeling below the fatigue limit.

### 3.2 Modeling of fatigue dispersion

The accumulation of fatigue damage is a typical random process, which can be expressed as Eq (3):

$$D(n)=F_{ati}(\{D_{i}\},\{D_{e}\},n)$$ (3)

where *D*_*i*_ represents the internal random variable, and it determines the randomness of damage, that is, the internal randomness;

*D*_*e*_ represents an external random variable, and it determines the randomness of damage, that is, the external randomness.

The inherent dispersion of material leads to significant dispersion of material fatigue life. According to the experiences in practice, the lognormal function can better represent the distribution of fatigue life of engineering parts. For example, suppose that the life *N* of material under constant amplitude stress *S* follows the logarithmic normal distribution with mean value of *μ*_*N*_ and variance of $\sigma_{N}^{2}$. Its probability density function is as shown in Eq (4):

$$f_{X}(x)=\left\{ \begin{array}{l} \frac{1}{\sqrt{2\pi }\sigma x}exp[-\frac{{\left(lnx-\mu \right)}^{2}}{2\sigma^{2}}]\;(x>0)\\ 0\;(x\leq 0) \end{array}\right.$$ (4)

The main numerical characteristics and distribution parameters of lognormal distribution are:

The mathematical expectation is as shown in Eq (5):

$$E(x)=exp\left[\mu +\frac{1}{2}\sigma^{2}\right]$$ (5)

The variance is as shown in Eq (6):

$$D(x)=exp[2\mu +\sigma^{2}](e^{\sigma^{2}}-1)$$ (6)

Material dispersion primarily arises from external factors, such as load and environment. Load randomness encompasses variations in both load magnitude and load sequence. When modeling fatigue randomness, a crucial step involves defining damage dispersion. Therefore, under constant amplitude loading, the damage under *n*_*i*_ loading cycles can be denoted by Eq (7):

$$D_{i}=\frac{n_{i}}{N_{i}}$$ (7)

where *N*_*i*_ represents fatigue life under constant amplitude loading, it is a random variable and $N∼Ln(\mu_{N},\;\sigma_{N}^{2})$.

Then, the distribution density function of instantaneous fatigue damage *D*(*n*) is as shown in Eq (8):

$$f_{D(n)}(x)=\left\{ \begin{array}{l} \frac{1}{\sqrt{2\pi }\sigma_{N}x}exp\left[-\frac{{\left(lnx-(-\mu_{N}+lnn)\right)}^{2}}{2\sigma_{N}^{2}}\right](x>0)\\ 0\;(x\leq 0) \end{array}\right.$$ (8)

Multi-level load is common in engineering. In our study, the two-level loading stresses are set as *S*_1_ and *S*_2_, respectively, and the loading times of *S*_1_ and *S*_2_ are *n*_1_ and *n*_2_. *E*_1_(*x*) and *E*_2_(*x*) are the expectations of generating damage when the two levels of stresses are loaded once, and *D*_1_(*x*) and *D*_2_(*x*) are the variances of generating damage when the two levels of stresses are loaded once. Under the combined effect of the two levels of stresses, set the expectation and variance of generating damage as *E*(*x*)_*D*(*n*)_ and *D*(*x*)_*D*(*n*)_, and we have the relations as shown in Eqs (9) and (10):

$${E(x)}_{D(n)}≅n_{1}{E(x)}_{1}+n_{2}{E(x)}_{2}$$ (9)

$${D(x)}_{D(n)}≅n_{1}{D(x)}_{1}+n_{2}{D(x)}_{2}$$ (10)

To sum up, the first and second moments of the lognormal distribution can be determined, that is, its *E*(*x*)_*D*(*n*)_ and *D*(*x*)_*D*(*n*)_. Then, the distribution parameters can be obtained by equivalence, as shown in Eqs (11) and (12):

$$\mu_{H}=ln\left(\frac{{E(x)}_{D(n)}}{\sqrt{\frac{{D(x)}_{D(n)}}{{E(x)}_{D(n)}^{2}}+1}}\right)=ln\left(\frac{n_{1}{E(x)}_{1}+n_{2}{E(x)}_{2}}{\sqrt{\frac{n_{1}{D(x)}_{1}+n_{2}{D(x)}_{2}}{{\left(n_{1}{E(x)}_{1}+n_{2}{E(x)}_{2}\right)}^{2}}+1}}\right)$$ (11)

$$\sigma_{H}^{2}=ln\left(\frac{{D(x)}_{D(n)}}{{E(x)}_{D(n)}^{2}}+1\right)=ln\left(\frac{n_{1}{D(x)}_{1}+n_{2}{D(x)}_{2}}{{\left(n_{1}{E(x)}_{1}+n_{2}{E(x)}_{2}\right)}^{2}}+1\right)$$ (12)

With the above equation, the instantaneous damage when the two levels of stresses are loaded can be equivalent to the lognormal distribution, and *μ*, *σ*^2^, and *D*(*n*)~ln(*μ*, *σ*^2^) can be obtained by mathematical expectation and variance when the instantaneous damage follows the lognormal distribution under the loading of two-level stresses. On this basis, it can be concluded that the instantaneous fatigue damage under multi-level loading still obeys the lognormal distribution, and its mathematical expectation and variance are denoted by Eqs (13) and (14):

$${E(x)}_{D(n)}≅n_{1}{E(x)}_{1}+n_{2}{E(x)}_{2}+\cdots +n_{k}{E(x)}_{k}=\overset{k}{\underset{i=1}{\sum }}n_{i}{E(x)}_{i}$$ (13)

$${D(x)}_{D(n)}≅n_{1}{D(x)}_{1}+n_{2}{D(x)}_{2}+\cdots +n_{k}{D(x)}_{k}=\overset{k}{\underset{i=1}{\sum }}n_{i}{D(x)}_{i}$$ (14)

Similarly, the distribution parameters after equivalence of the lognormal distribution under multi-stage loading are denoted by Es (15) and (16):

$$\mu_{H}=ln\left(\frac{{E(x)}_{D(n)}}{\sqrt{\frac{{D(x)}_{D(n)}}{{E(x)}_{D(n)}^{2}}+1}}\right)=ln\left(\frac{\overset{k}{\underset{i=1}{\sum }}n_{i}{E(x)}_{i}}{\sqrt{\frac{\overset{k}{\underset{i=1}{\sum }}n_{i}{D(x)}_{i}}{{\left(\overset{k}{\underset{i=1}{\sum }}n_{i}{E(x)}_{i}\right)}^{2}}+1}}\right)$$ (15)

$$\sigma_{H}^{2}=ln\left(\frac{{D(x)}_{D(n)}}{{E(x)}_{D(n)}^{2}}+1\right)=ln\left(\frac{\overset{k}{\underset{i=1}{\sum }}n_{i}{D(x)}_{i}}{{\left(\overset{k}{\underset{i=1}{\sum }}n_{i}{E(x)}_{i}\right)}^{2}}+1\right)$$ (16)

The critical value of fatigue damage will follow the same distribution due to the dispersion of fatigue life. In this section, the dispersion of fatigue life has been considered, so the critical value of fatigue damage *D*_*cr*_ is set as constant 1. Moreover, the stiffness requirements of component should also be considered when predicting the life. The fatigue damage defined by stiffness is as shown in Eq (17):

$$D_{cr}=1-\frac{K_{min}}{K_{0}}$$ (17)

where, *K*_*min*_ represents critical value of stiffness. *K*_0_ represents initial stiffness; *D*_*cr*_ represents critical value of damage that satisfies the stiffness requirements.

### 3.3 Damage modeling under fatigue limit

Set *σ*_*e*_ as the fatigue limit of the test piece, and then, the membership function for fatigue prediction is as defined in Eq (18).

$$\mu (x)=\left\{ \begin{array}{l} 1\;,(\sigma_{i}\geq \sigma_{e})\\ \frac{N_{0}}{N_{i}},(\sigma_{i}<\sigma_{e}) \end{array}\right.$$ (18)

where, *N*_0_ represents the base number of stress cycles, i.e., the number of cycles corresponding to the fatigue limit.

According to the definition of membership function, when the cyclic stress is greater than the fatigue limit, membership 1 denotes that it will cause damage; when the cyclic stress is smaller than the fatigue limit, $\frac{N_{0}}{N_{i}}$ is used to represent the damage caused by different stresses, and generally *N*_*i*_>*N*_0_.

The fuzzy fatigue life model is determined by examining the relationship between cyclic stress and fatigue limit. If the cyclic stress is relatively small, the membership degree of change in the definition of the membership function is utilized to establish the model. Conversely, when the cyclic stress is large, the life model is determined based on the traditional Miner theory. By employing this comprehensive approach, a fuzzy fatigue life model can be derived, as demonstrated in Eq (19).


$$N=\left\{ \begin{array}{l} {\left(\frac{\sigma_{e}}{\sigma }\right)}^{m}N_{0},(\sigma_{i}\geq \sigma_{e})\\ \frac{N_{0}}{\mu (x)}\;,(\sigma_{i}<\sigma_{e}) \end{array}\right.$$ (19)

Assuming a certain stress spectrum, there are *m* stress levels, each stress level of the actual number of actions is *n*, in which the stress greater than the fatigue limit has *k* poles, and the total damage of the test piece during fatigue failure is represented by *D*, then Eq (20) can be obtained:

$$D=\sum_{i=1}^{k}\frac{n_{i}}{N_{i}}+\sum_{j=k+1}^{n}\frac{n_{j}}{N_{0}}\mu (x)$$ (20)

where, $\overset{k}{\underset{i=1}{\sum }}\frac{n_{i}}{N_{i}}$ represents fatigue damage caused by cyclic stress higher than the fatigue limit. $\overset{n}{\underset{j=k+1}{\sum }}\frac{n_{j}}{N_{0}}\mu (x)$ represents fatigue damage caused by stress lower than the fatigue limit determined by the membership function.

In Eqs (19) and (20), *μ*(*x*) is the membership function. A large-scale membership function is often used to analyze the fuzziness of fatigue damage. The commonly used large-scale membership functions in engineering include the following three types:

a. With large semi-trapezoidal distribution, as shown in Eq (21).


$$\mu (x)=\left\{ \begin{array}{l} 0,\;(x\leq a_{1})\\ \frac{x-a_{1}}{a_{2}-a_{1}},(a_{2}>x>a_{1})\\ 1,\;(x\geq a_{2}) \end{array}\right.$$ (21)

b. With large normal distribution, as shown in Eq (22).


$$\mu (x)=\left\{ \begin{array}{l} 0,\;(x\leq a)\\ 1-e^{{-(\frac{x-a}{a})}^{2}},(x>a) \end{array}\right.$$ (22)

c. With large parabolic distribution, as shown in Eq (23).


$$\mu (x)=\left\{ \begin{array}{l} \;0,\;(x\leq a_{1})\\ {\left(\frac{x-a_{1}}{a_{2}-a_{1}}\right)}^{2},(a_{2}>x>a_{1})\\ \;1,\;(x\geq a_{2}) \end{array}\right.$$ (23)

**Fig 3:**
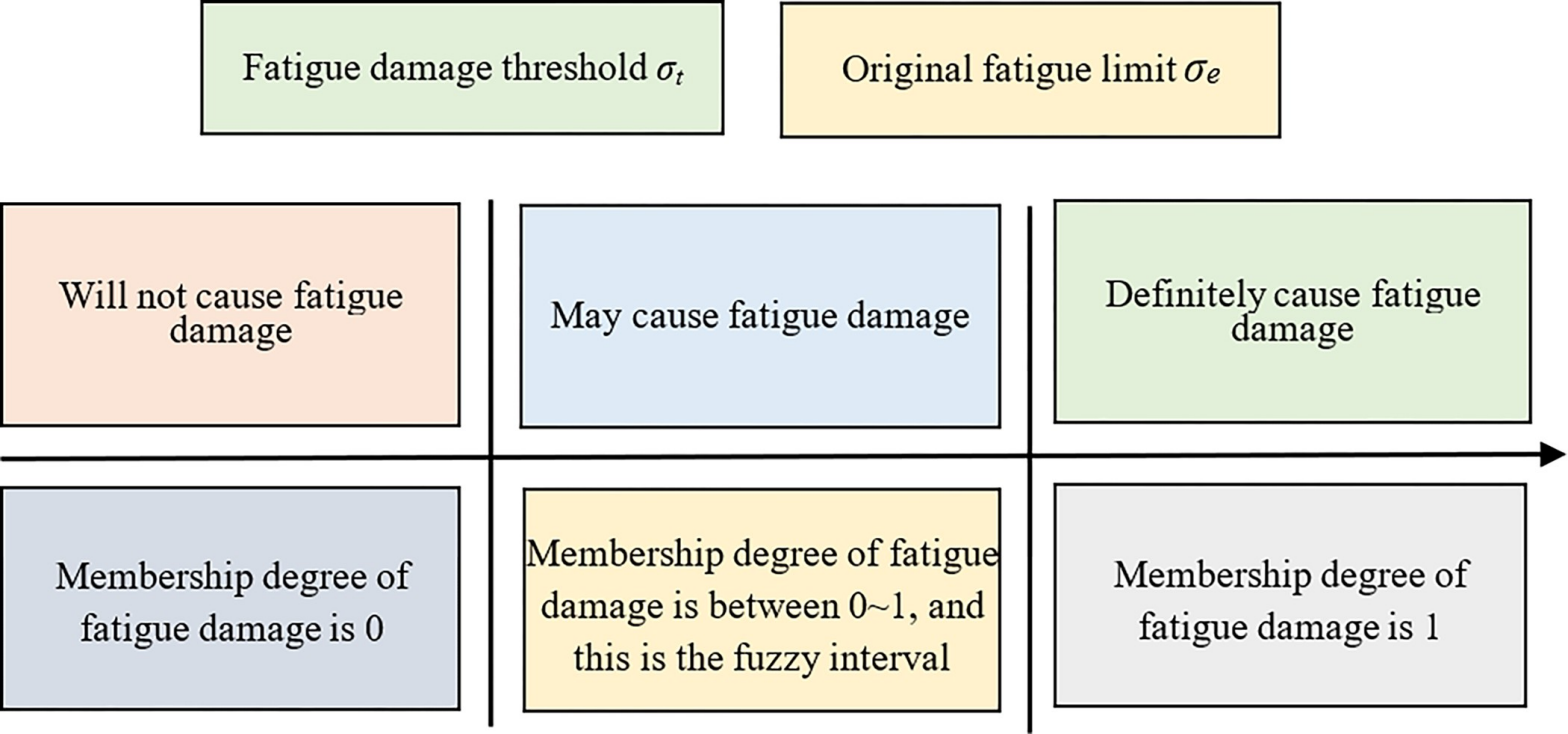
Fuzzy area of fatigue damage.

The relationship between the "fuzzy areas" of fatigue limit and fatigue damage is often employed to depict the influence of load on component fatigue life. Generally, the fatigue life corresponding to a loading sequence from high to low is lower than that of a sequence from low to high, owing to the tendency of the latter sequence to generate the "induced reinforcement effect." The structure of the former sequence exhibits the relationship between the "fuzzy areas" of fatigue limit and fatigue damage, as illustrated in Fig 3. In order to determine the fuzzy interval, a key step is to determine the fatigue damage threshold *σ*_*t*_, and relatively accurate prediction results can be obtained when *σ*_*t*_ is between 0.3*σ*_*e*_~0.4*σ*_*e*_.

Combining the above equations, set *N*_*f*_ as the fatigue life of the test piece, and it is easy to get the fuzzy fatigue life prediction model as shown in Eq (24).


$$N_{f}=\frac{\left(\overset{k}{\underset{i=1}{\sum }}n_{i}+\overset{n}{\underset{j=k+1}{\sum }}n_{j}\right)}{D}$$ (24)

$$N_{f}=\frac{\overset{k}{\underset{i=1}{\sum }}\frac{n_{i}}{N_{i}}}{D}$$ (24)

### 3.4 Simulation analysis

In this study, a comprehensive analysis model of the finite corner motor and eccentric drive mechanism was initially established using SolidWorks software, which was subsequently imported into ANSYS for analysis. The specific analysis process is as follows:

1. Determining the structural analysis scheme: Workbench offers various analysis methods tailored to different solid structures.
2. Defining the material properties: ANSYS Workbench allows users to select relevant materials from the material database or customize material properties to suit specific requirements.
3. Creating analytical geometry models: Workbench provides two modeling approaches. One involves building the geometric model in external 3D modeling software and importing it into Workbench, while the other allows for direct creation of geometric models using the available modeling functions within Workbench.
4. Establishing the finite element model: To enhance the accuracy and smoothness of the finite element analysis, an appropriate meshing method is employed. The software offers various meshing techniques such as the multi-domain method, scanning method, and automatic division method, which allow for the creation of tetrahedral or hexahedral meshes based on the specific analysis requirements.
5. Applying constraints and loads: Workbench provides a range of constraint types and load forms that can be selected according to the actual force conditions experienced by the object under analysis.
6. Analysis setup and solution: Proper solution options and result items are set, enabling the viewing and evaluation of the solution results.

In summary, the finite element simulation conditions employed in this study were as follows: The structural analysis utilized the Solid178 unit. The valve body was treated as a homogeneous material with an elastic modulus E = 200GPa and a Poisson’s ratio μ = 0.30. To account for gravity effects, a density ρ = 7.85kg/m^3^ and a gravitational acceleration of 9.8m/s^2^ were considered. The intelligent grid division was set to the finest level, resulting in 13,482 nodes for the eccentric drive mechanism. The overall cell size was controlled to be 1 mm. Additionally, there were 21,345 nodes for the finite corner motor and 13,482 nodes for the eccentric drive mechanism. For the application of constraints and loads, full constraints were applied to the finite corner motor section, while certain pressure loads were applied to the contact surface between the finite corner motor and the eccentric drive mechanism. The FEA solver selected for this valve was the preconditioned common choke gradient (PCG) method.

**Fig 4:**
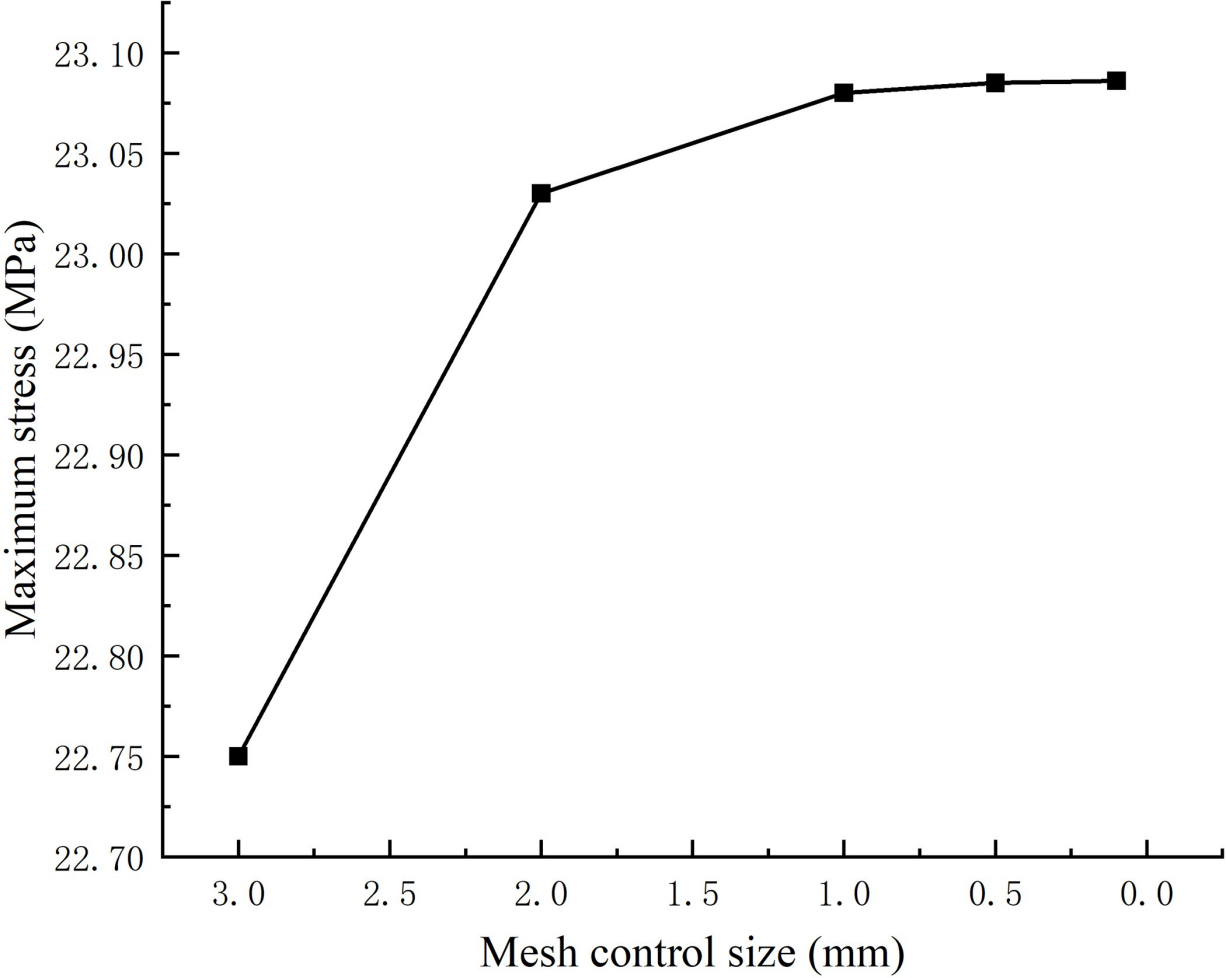
Mesh control size and maximum stress relationship diagram.

In the division of grid elements, the principle of "coarse division for regions of uniform stress and fine division for regions with large stress gradients" is adhered to. Specifically, for valve torque motor components, fine meshing is required in the eccentric drive mechanism and its connection to the finite angle motor. Conversely, the remaining areas of the finite angle motor are divided into coarse grids. To verify grid independence, the overall element size is controlled at 3 mm, 2 mm, 1 mm, 0.5 mm, and 0.1 mm, respectively. The maximum stress variation under different grid settings is illustrated in Fig 4. The results indicate that when the overall element size is controlled within 1 mm, the maximum equivalent stress value stabilizes as the mesh size decreases. Considering computational resources, the overall element size is set to 1 mm. At this size, the finite angle motor has 21,345 nodes, and the eccentric drive mechanism has 13,482 nodes.

**Fig 5:**
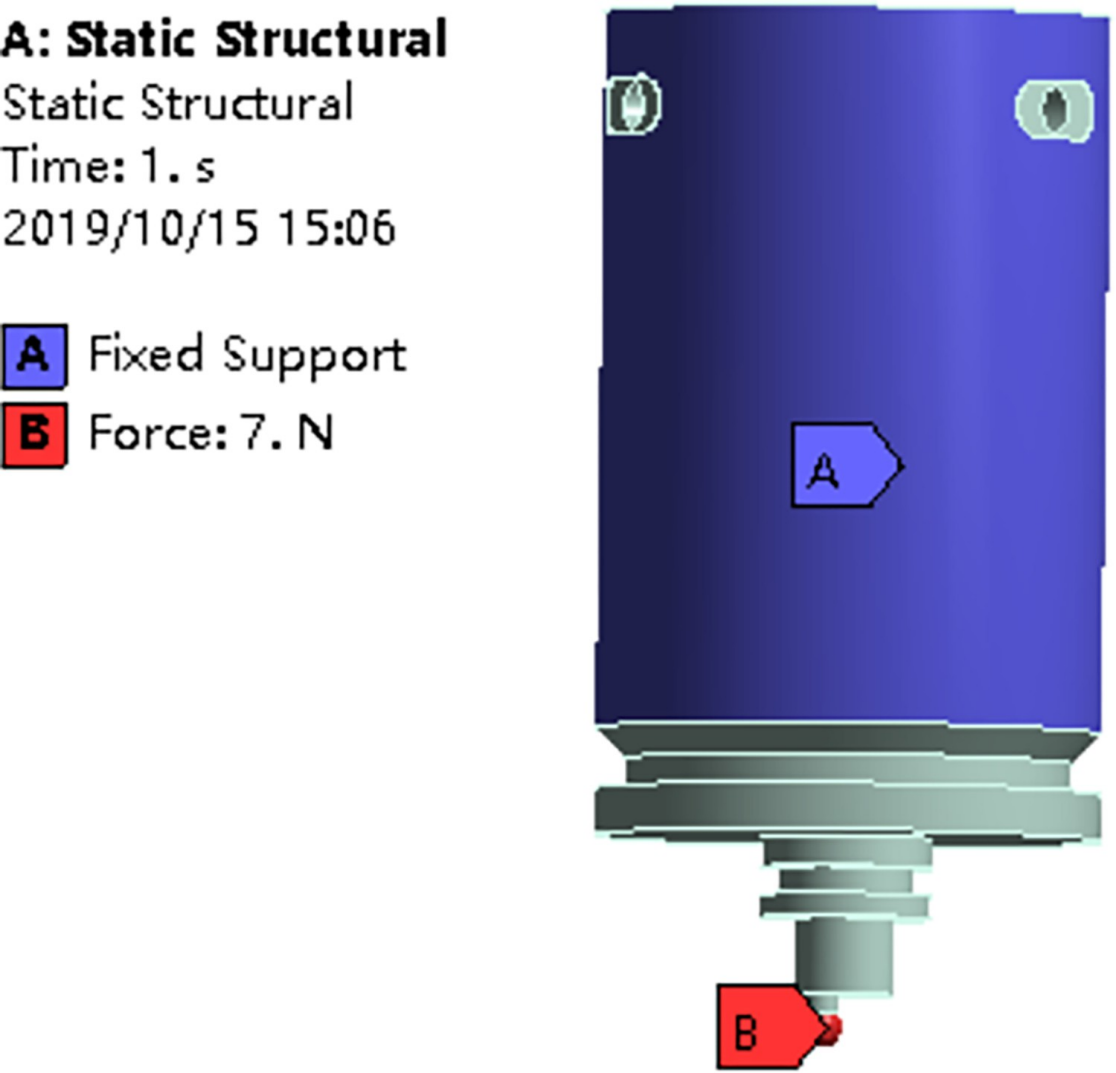
Boundary conditions of eccentric drive mechanism.

**Fig 6:**
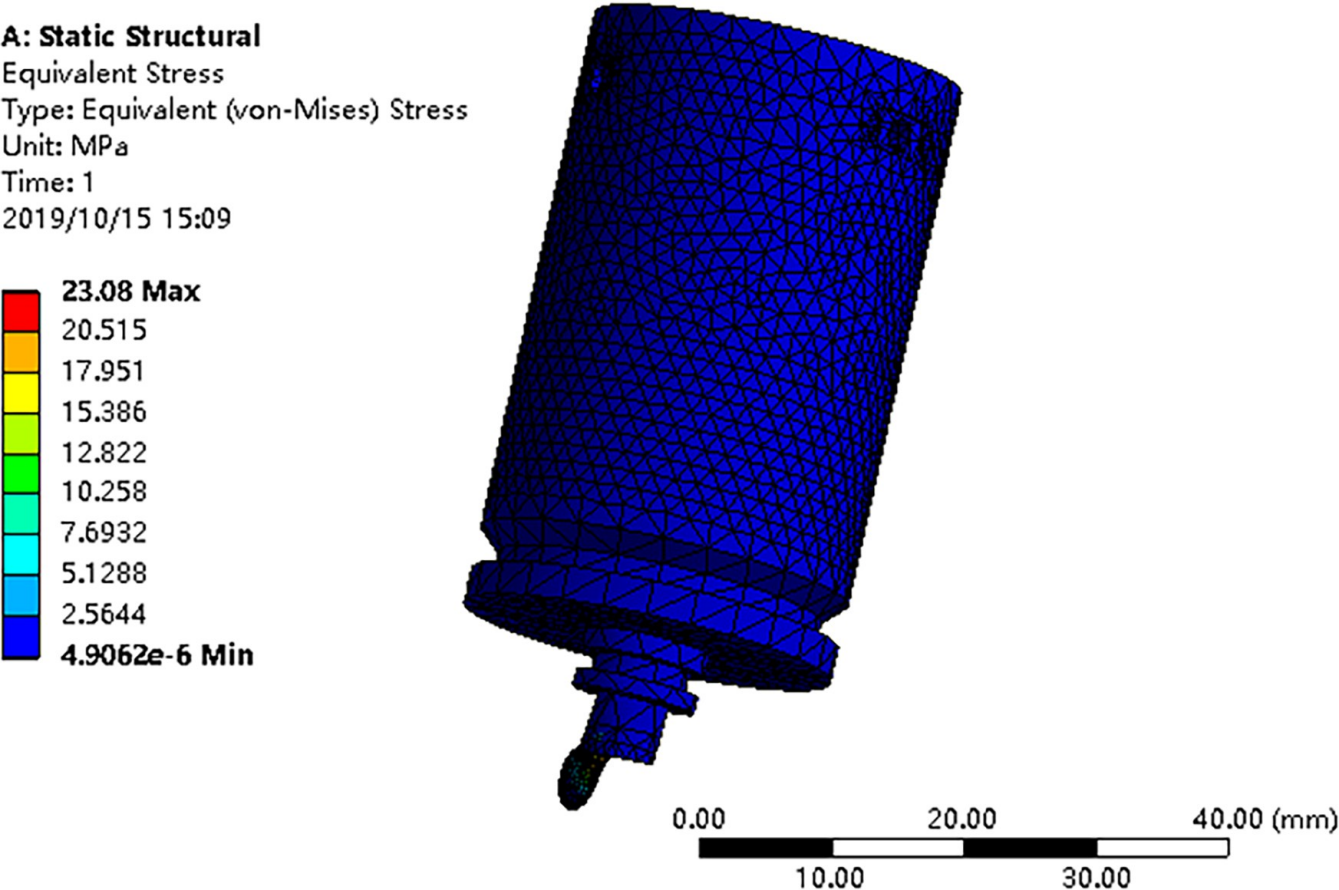
Stress program of eccentric drive mechanism.

This section predicts the durability life of the eccentric drive mechanism of the torque motor based on stiffness requirements, utilizing the fatigue dispersion model and the damage model under the fatigue limit. In practical engineering, the eccentric drive mechanism experiences high-cycle fatigue characterized by relatively low cyclic stress levels and primarily elastic deformation. To simulate the behavior of the eccentric drive mechanism under actual working conditions, ANSYS software is employed. The fatigue life prediction model, based on the membership function, is then used to estimate the component’s fatigue life. Considering the internal and external material variations in the eccentric drive mechanism, the moment equivalent probability method is applied to predict the dispersion of fatigue life. The internal dispersion is based on fatigue test data obtained from 45 steel. The material performance parameters for the eccentric drive mechanism are presented in Table 2. Following the actual engineering conditions, the ball of the eccentric drive mechanism is constrained, and a force load of 7N is applied in the direction of spool movement. Rigid connections are established for the assembly’s contacts, with mesh refinement applied to key components. The boundary conditions of the eccentric drive mechanism are illustrated in Fig 5, respectively. The cyclic stress amplitude is analyzed, and the resulting stress distribution is shown in Fig 6. Using the "Insert/Equivalent" option in the software, relevant parameters are set for equivalent stress simulation. The maximum stress, approximately 23.08MPa, is observed at the top of the ball. Notably, the stress levels on the eccentric drive mechanism remain below the minimum fatigue limit of 255MPa for the selected material, 45 steel.

**Table 2:** Material performance parameters of the eccentric drive mechanism.

Component name	Material	Elasticity modulus *E*/GPa	Poisson’s ratio *μ*	Density *ρ/*(kg·m^-3^)
Eccentric drive mechanism	45# steel	200	0.30	7850

### 3.5 Calculation of durability indicators

In the prediction of the durability life for the eccentric drive mechanism, as the input parameters of the pressure valve are gradually reduced, we observe a decrease in the maximum stress amplitude. However, the maximum stress value remains at the bottom of the eccentric drive mechanism, but it is only 23.08 MPa. In cases where fuzzy damage rules are unclear, it is preferable to use a membership function with a large semi-trapezoidal distribution and a simple expression. Generally, high prediction precision can be achieved when *σ*_*t*_ is within the range of 0.30*σ*_*e*_ to 0.40*σ*_*e*_. During an in-depth investigation of the problem, continuous modifications can also be made. In our study, we set *σ*_*t*_ = 0.30*σ*_*e*_ to determine the fatigue life dispersion of the eccentric drive mechanism. However, since the maximum stress is significantly smaller than 0.30*σ*_*e*_ [29], we can ignore the fatigue damage in the eccentric drive mechanism, indicating that it has reached its permanent fatigue life.

## 4. Wear durability analysis of slide valve

**Fig 7:**
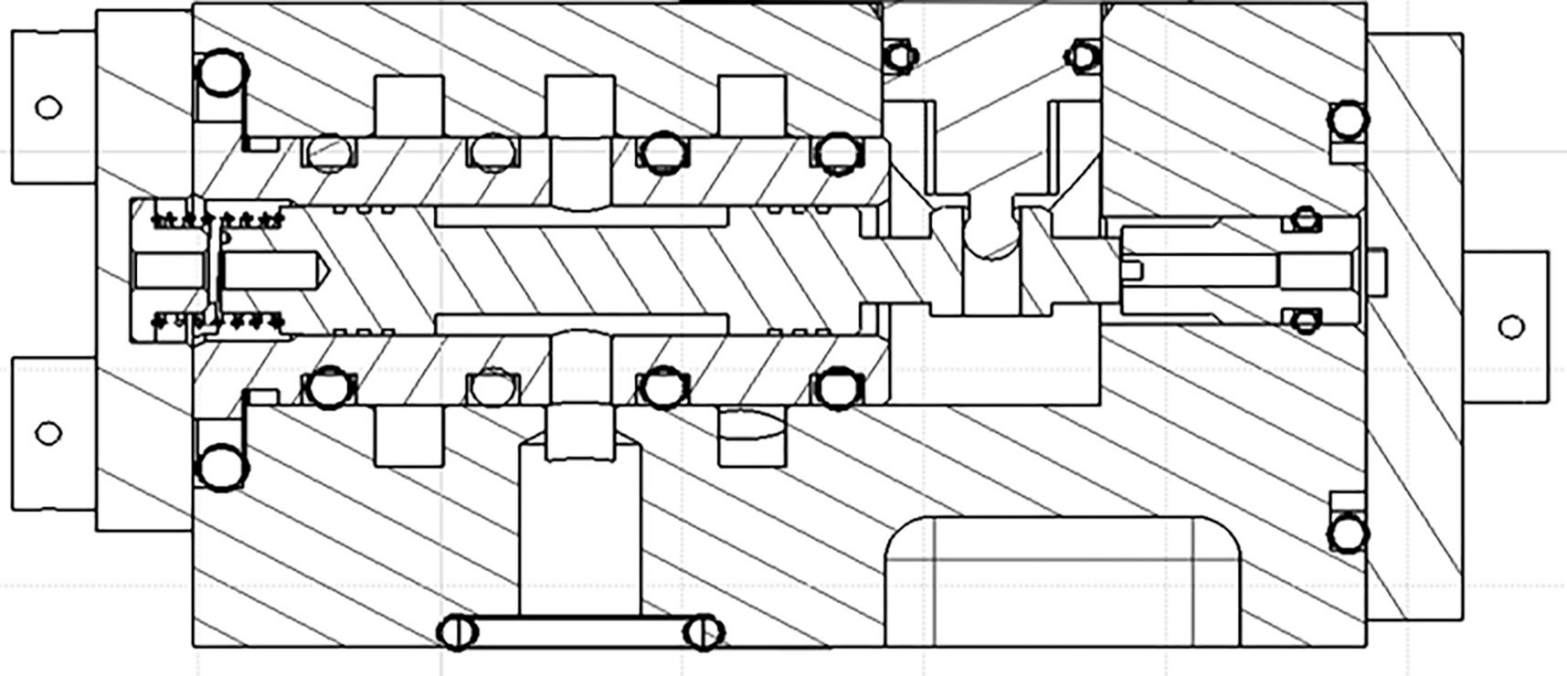
Structure diagram of RDDPV slide valve pair.

As shown in Fig 7, the RDDPV slide valve pair consists of the spool and the valve sleeve. The reciprocating motion of the spool corresponding to the valve sleeve will generate wear, which will increase the radial clearance between the spool and the valve sleeve.

**Fig 8:**
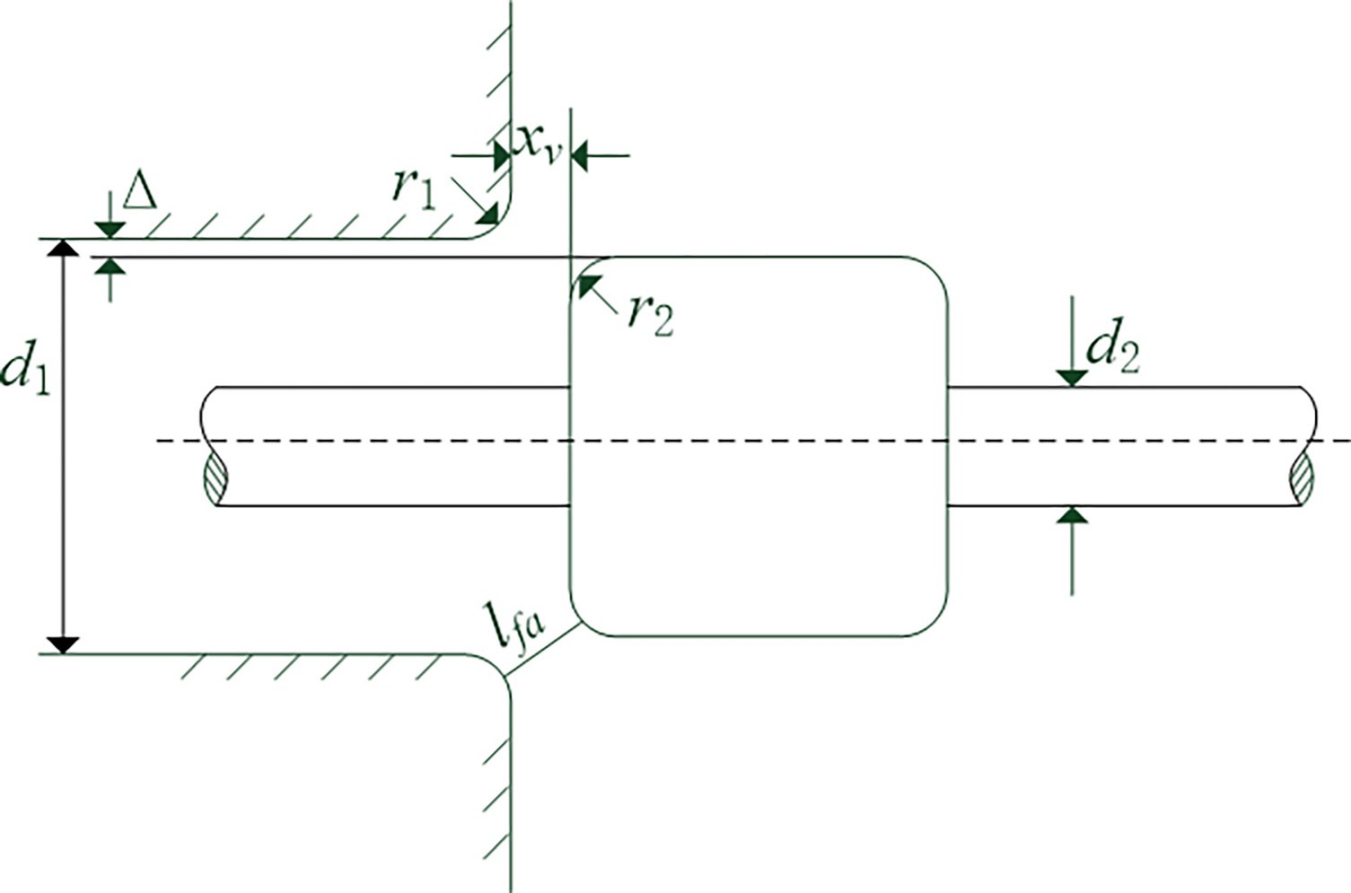
Model of the slide valve orifice.

Wear results in an increase in the radial clearance Δ, as depicted in Fig 8. Consequently, the equivalent opening for the same spool displacement *x*_*v*_ becomes larger, leading to a significant rise in the flow coefficient of the throttle orifice within the small opening range. However, as the spool displacement continues to increase, the flow coefficient stabilizes at a constant value. The calculation for determining the equivalent opening is presented in Eq (25).


$$l_{fa}=\sqrt{{\left(r_{1}+r_{2}+\Delta \right)}^{2}+{\left(x_{v}+r_{1}+r_{2}\right)}^{2}}-(r_{1}+r_{2})$$ (25)

### 4.1 Durability analysis and modeling

#### 4.1.1. Analysis of the critical value of failure

The radial clearance between the spool and the valve sleeve significantly influences the performance of the valve [30]. Based on the results of static and dynamic simulation analysis, it is observed that when the radial clearance between the spool and the valve sleeve reaches 5.16 μm, the valve performance deteriorates to a critical failure point. Considering the initial clearance between the spool and sleeve is 3 μm, the permissible wear value is *h*_*max*_ = 2.16*μm*。

#### 4.1.2. Modeling of failure rules

In this project, both the spool and the valve sleeve are fabricated from the same material. The contact between the spool and valve sleeve results in high pressure, leading to adhesive wear of the metal [31]. Alongside this wear, abrasive wear also occurs between the spool and sleeve. To analyze this intricate wear phenomenon, Professor Kraguelsky proposed a wear process based on linear wear degree, illustrating the relationship between macro and micro characteristics. These relationships are depicted in Eqs (26) and (27) respectively [30, 31].

$$I_{h}=\frac{\Delta H}{L_{T_{p}}}=\frac{\bar{d_{1}}\bar{d_{2}}}{\Delta \bar{A_{r}}}\frac{\Delta h}{\bar{d_{2}}}\frac{A_{r}}{A_{a}}$$ (26)

$$i_{h}=\frac{\Delta h}{\bar{d_{2}}}=\frac{\Delta V}{A_{r}\bar{d_{2}}}$$ (27)

where, *I*_*h*_ is the wear degree; *I*_*h*_ is the unit wear degree; Δ*H* is linear wear/m; $L_{T_{p}}$ is the relative stroke of wear/m; Δ*h* is the assumed wear/m; $\bar{d_{1}}$ is the average size of the micro-spots of wear in the vertical direction to the sliding direction/m; $\bar{d_{2}}$ is the average size of the micro-spots of wear in sliding direction/m; *A*_*r*_ is the actual contact area of friction/m^2^; Δ*A*_*r*_ is the average area of a single contact spot/m^2^; *A*_*a*_ is the nominal contact area/m^2^; Δ*V* is the volume change/m^3^.

When two wear surfaces contact, assume that the starting segment of the support surface curve can be expressed by Eq (28).


$$t^{P}=bx^{v}$$ (28)

Then, the compression volume *V*_*B*_ is:

$$V_{B}=A_{C}R_{max}b\int_{0}^{\varepsilon }x^{v}dx=\frac{A_{C}b\varepsilon^{v+1}R_{max}}{v+1}=\frac{\varepsilon }{v+1}R_{max}A_{r}$$ (29)

where, *ε* is the proximity between two objects/m; *A*_*C*_ is the contact area of friction profile/m^2^; *R*_*max*_ is the maximum height of profile unevenness, *A*_*r*_ is the actual contact area of friction/m^2^; *b* and *v* are related parameters of profile.

During the wear process, there is a period of latency before the abrasive particles dislodge as a result of interactions between convex bodies. To account for this, a coefficient *n* is introduced to quantify the number of actions necessary for the formation of abrasive particles. Consequently, the unit wear degree and macro wear degree can be expressed using Eqs (30) and (31) respectively:

$$i_{h}=\frac{\varepsilon }{(v+1)n}\frac{R_{max}}{\bar{d}}\;$$ (30)

$$I_{h}=\frac{b\varepsilon^{v+1}R_{max}}{(v+1)n\bar{d}}\frac{A_{r}}{A_{a}}=\frac{b\varepsilon^{v+1}R_{max}}{(v+1)n\bar{d}}\eta_{c,a}$$ (31)

where, $\bar{d}$ is the diameter of micro-convex body/m, $\bar{d}=\bar{d_{1}}=\bar{d_{2}}$.

Under plastic compression, its deformation is as shown in Eq (32):

$$\varepsilon ={\left(P_{c}/c\sigma_{T}b\right)}^{1/v}$$ (32)

If the profile of the contact spot is approximated to a circumference, then the contact spot area $\bar{\Delta A_{r}}$ and the contact spot radius $\bar{d_{n}}$ are as shown in Eqs (33) and (34), respectively:

$$\bar{\Delta A_{r}}=\frac{2\pi rR_{max}}{v}\varepsilon =\frac{2\pi r^{2}}{v}\Delta {\left(\frac{p_{c}}{c\sigma_{T}}\right)}^{1/v}$$ (33)

$$\bar{d_{n}}=2\sqrt{2}{\left(\frac{\pi rR_{max}}{v}\varepsilon \right)}^{1/2}=\frac{2\sqrt{2}r}{\sqrt{v}}\Delta^{1/2}{\left(\frac{p_{c}}{c\sigma_{T}}\right)}^{1/2v}$$ (34)

where, *σ*_*T*_ is the yield limit of material/Pa; Δ is the roughness parameter.

According to the linear cumulative damage theory and the mechanical friction characteristics at a single contact point, the number of failure cycles *n* can be obtained, as shown in Eq (35).

$$n={\left(\frac{2re_{0}}{\bar{d_{n}}}\sqrt{\frac{\sigma_{T}-2fHB}{(\sigma_{T}+2fHB)v}}\right)}^{t}K_{tv}$$ (35)

where, *e*_0_ and *t* are the friction fatigue curve parameters; *f* is the friction coefficient; HB is Brinell hardness/N·mm^-2^; *K*_*tv*_ is the introduced correction coefficient.

Combining Eqs (31), (32), (34), and (35), we can obtain the linear wear degree *I*_*h*_, as shown in Eq (36):

$$I_{h}=\sqrt{2}C\left(\frac{p_{a}}{HB}\right)\frac{1+\beta t}{1-\beta }\Delta^{\frac{1+t}{2}}{\left(\frac{K_{t}^{n}}{e_{0}}\right)}^{t}{\eta_{c,a}}^{-\frac{1+\beta t}{1-\beta }}$$ (36)

where, *β* = 1/(2*v*+1); $K_{t}^{n}={\left(\sigma_{T}+2fHB\right)}^{1/2}/{\left(\sigma_{T}-2fHB\right)}^{1/2}$; *p*_*a*_ is the positive pressure/N, *C* is the fatigue limit.

Another commonly applied form of Eq (36) is presented in Eq (37):

$$I_{h}=K_{1}K_{t}\alpha {\left(\frac{1}{2}\right)}^{t_{y}-1-\frac{1}{\xi }}\sigma_{H}^{1+\frac{t_{y}}{2\xi +1}}\lambda^{\frac{\xi t_{y}}{2\xi +1}}E^{\frac{2\xi t_{y}}{2\xi +1}-1}{\left(\frac{kf_{M}}{\sigma_{0}}\right)}^{t_{y}}$$ (37)

where, *K*_1_ represents the coefficient determined by the distribution along the height of the geometric profile of the material surface, and *K*_*t*_ represents the correction factor. Area ratio, *α = A*_*a*_/*A*_*r*_, *A*_*a*,_ and *A*_*r*_ are the nominal contact area and actual contact area. *σ*_*H*_ represents average contact stress/Pa. *ξ* represents the parameter of the material micro surface support curve. *t*_y_ represents the parameter of the material friction fatigue curve. *λ* represents comprehensive characteristics of surface roughness. *k* represents the contact stress state coefficient. *f*_*M*_ represents the friction coefficient. *σ*_0_ represents the ultimate failure stress of material/Pa. *E* represents elasticity modulus/Pa.

### 4.2 Calculation of durability indicators

**Fig 9:**
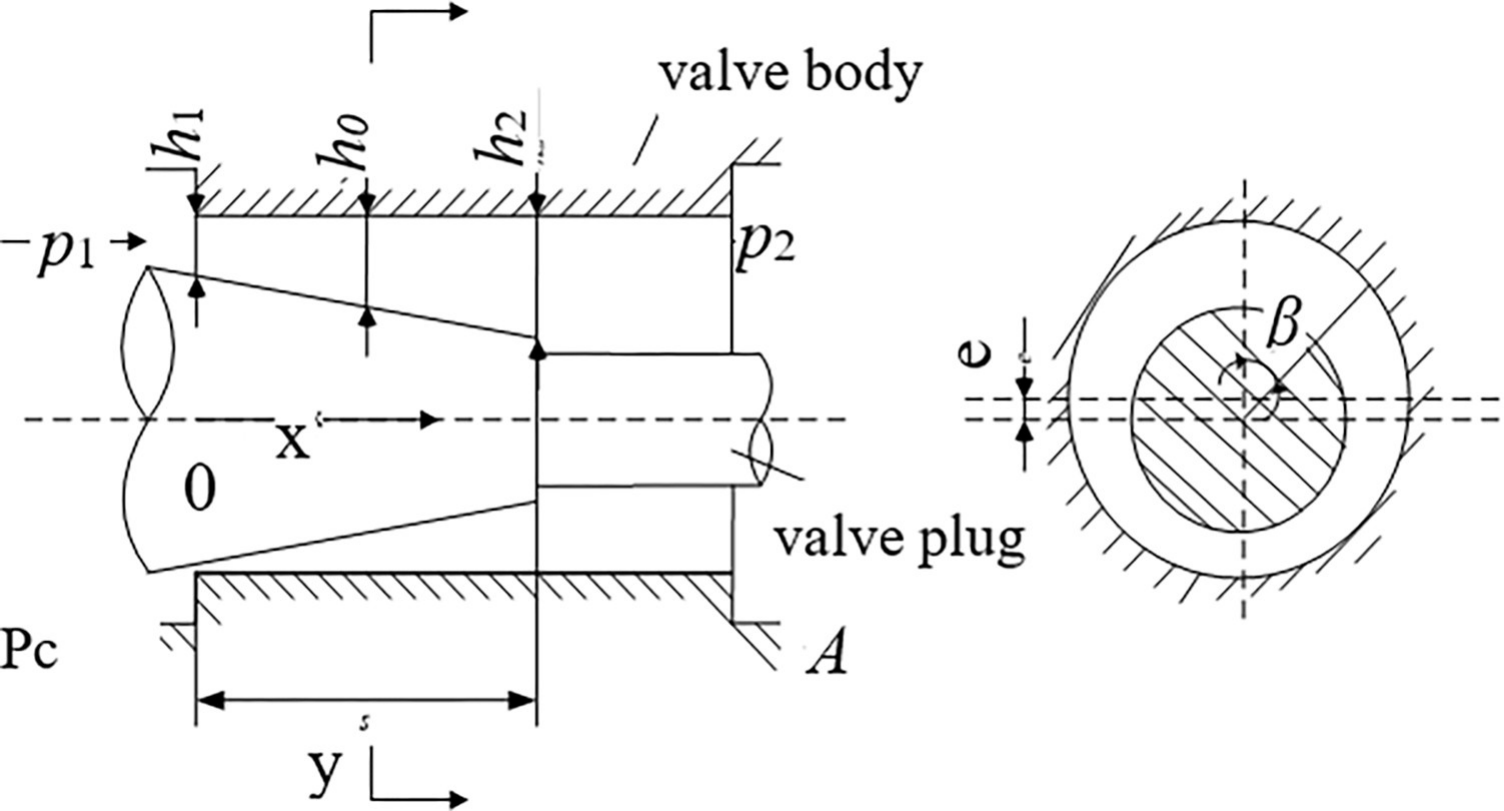
Partial enlarged view of RDDPV wear.

In this section, we employ the linear wear degree to quantify the radial wear occurring between the valve spool and valve sleeve. In practical applications, machining errors inevitably result in the formation of a tapered conical surface on the spool. Consequently, upon installation of the spool into the valve sleeve, an annular clearance flow is generated between the inner conical surface and the outer cylindrical surface. If this annular clearance flow exhibits divergence, it gives rise to a lateral force. The average contact stress between the valve spool and the valve sleeve is thus influenced by this lateral force acting at the shoulder of the spool. Fig 9 illustrates a simplified model of the first shoulder at the fitting section between the spool and sleeve. Within the figure, *p*_1_ and *p*_2_ represent the pressures at the larger and smaller ends of the cone, while *h*_1_ and *h*_2_ denote the clearances at these respective ends. Additionally, the radius difference between the larger and smaller ends of the inverted cone, determined by the maximum cylindricity deviation, is 0.0008 mm.

The pressure at the clearance *h*_0_ of the fitting section is as shown in Eq (38).

$$P_{0}=P_{1}-(P_{1}-P_{2})\frac{{\left(\frac{h_{1}}{h_{0}}\right)}^{2}-1}{{\left(\frac{h_{1}}{h_{2}}\right)}^{2}-1}$$ (38)

where, $h_{1}=h_{01}+e\;\cos\;\beta =0.003+0.003\;\cos\;\beta ,\;h_{2}=h_{02}+e\;\cos\;\beta =0.0055+0.003\;\cos\;\beta ,\;{and\;h}_{0}=h_{1}+\frac{(h_{2}-h_{1})x}{L}=0.003+0.003\;\cos\;\beta +0.00045x$.

Then, the lateral force is as shown in Eq (39):

$$F=\int_{0}^{2\pi }f\times \frac{d}{2}\cos\;\beta \;d\beta =\int_{0}^{2\pi }(\int_{0}^{L}p_{0}dx)\times \frac{d}{2}\cos\;\beta \;d\beta =25.70N$$ (39)

where, *p*_1_ represents oil supply pressure, 21MPa. *p*_2_ represents oil return pressure, 0.5MPa. *e* represents the full eccentricity of the spool, *e* = 0.003mm. *d* represents the big-end diameter of the spool, *d* = 6mm. Similarly, the lateral force at the other shoulder of the spool can be calculated, which is 26.40N. According to related engineering practices, the influence factor of slotting three pressure-equalizing grooves on the shoulder is 0.06, so when there is no offset of the spool, it is under the lateral force *F* of 3.13N. In this case, the contact area *S* between the spool and sleeve is 32.17mm^2^, so the contact stress is as shown in Eq (40):

$$\sigma_{H}=\frac{F}{S}=0.097MPa$$ (40)

Substitute it into Eq (37). Then, by combining the actual working conditions of the valve, and using the parameters listed in Table 3, the single opening wear of the valve spool can be obtained, as shown in Eq (41):

$$h=\int_{0}^{s}K_{1}\;K_{t}\alpha {\left(\frac{1}{2}\right)}^{t_{y}-1-\frac{1}{\xi }}\sigma_{H}^{1+\frac{t_{y}}{2\xi +1}}\lambda^{\frac{\xi t_{y}}{2\xi +1}}E^{\frac{2\xi t_{y}}{2\xi +1}-1}{\left(\frac{kf_{m}}{\sigma_{0}}\right)}^{t_{y}}ds=1.2430\times 10^{-12}mm$$ (41)

where, *s* represents relative movement stroke, *s* = 0.1mm.

**Table 3:** Values of parameters related to linear wear degree.

Parameter	Value	Parameter	Value
*K*_t_	2	*k*	3
*f*_m_	0.1	*K*_1_	0.2
*t*_y_	7.9	*E*(MPa)	2.11×10^5^
*λ*	2.2×10^−2^	*σ*_0_(Pa)	1000
*α*	1	*ζ*	1.5

When the wear clearance between the valve spool and sleeve reaches *h*_max_/2 = 1.08μm, the operating times the spool is its durability wear life, which is presented in Eq (42):

$$M=\frac{h_{max}}{2h}=8.7\times 10^{8}\;$$ (42)

The durability analysis provides results for the identified vulnerable components of the rotary direct drive pressure valve, expressing their service life in "cycles". To facilitate comparison with the service life of other pressure valves, it becomes necessary to convert between the two units of "cycles" and "hours". The conversion coefficient primarily relies on the specific working conditions. By employing a conversion rate of 2000 cycles per hour, the service lives of individual parts and the entire valve can be determined, as presented in Table 4.

**Table 4:** Failure time of whole valve and components.

Component	Failure mechanism	Failure time/h	Valve life/h
Spring tube	Stress fatigue	Permanent life	435000
Valve spool and sleeve	Adhesive wear	435000	

## 5. Results and discussion

In this study, a comprehensive analysis was conducted to ascertain the durability and prognosis of the operational lifespan of a rotary direct-drive electrohydraulic servo valve (RDDPV). A durability analysis method rooted in the principles of failure physics was proposed, encompassing exhaustive fatigue durability analysis of the transmission mechanism and wear durability analysis of the slide valve.

For the analysis of the transmission mechanism, a novel fatigue life prediction approach amalgamating probabilistic and fuzzy theories with traditional Miner theory was introduced to dissect the fatigue life transfer mechanism. This encompassed both fatigue dispersion modeling and damage modeling at the fatigue limit. Utilizing ANSYS 17.0 software, the cyclic stress amplitude of the eccentric transmission mechanism under authentic operational conditions was simulated and meticulously analyzed. Employing a fatigue life prediction model based on the fatigue life prediction function, the fatigue life of the components was estimated. Furthermore, fatigue life dispersion prediction was executed using the method of moment-equivalent probability, integrating intrinsic dispersion derived from fatigue test data of 45 steel. The analysis revealed that the stress value in the eccentric drive mechanism remained below the minimum fatigue limit of the chosen material, 45 steel, recorded at 255 MPa.

For the analysis of the slide valve, quantification of the radial wear value between the slide valve sleeves was accomplished utilizing the linear wear degree. Through the establishment of failure law models and subsequent durability index calculations, an overall valve life estimate of 435,000 hours was determined. The calculated durability life values satisfyingly adhere to the national standard requirement of 5,000 hours or 10^7^ cycles of service life for jet pipe servo valves.

## 6. Conclusion

The research methodology and findings presented in this paper serve as a theoretical foundation for the forthcoming reliability analysis and pollution resistance evaluation of RDDPVs. Additionally, these results hold significant reference value for qualitative analysis, predictive modeling of durability in electro-hydraulic servo valves of a similar kind, and quantitative assessments of their operational lifespan.

The electro-hydraulic pressure servo valve, developed following the principles delineated in this study, presents a viable alternative to the present employment of jet pipe pressure servo valves and nozzle-baffle pressure servo valves in aircraft. This novel valve not only inherits the robust anti-pollution capabilities of jet pipe pressure servo valves but also addresses the inherent issue of static consumption of flow that plagues such valves. Consequently, it substantially enhances hydraulic energy utilization efficiency. Furthermore, this valve design incorporates a versatile multi-signal control interface, facilitating convenient monitoring of the valve’s operational status and enabling enhanced control for users.
